# Ischémie mésentérique aiguë veineuse chez un jeune sujet COVID-19 positif: à propos d’un cas

**DOI:** 10.11604/pamj.2021.39.273.30454

**Published:** 2021-08-26

**Authors:** Oussama Marsafi, Fadoua Ijim, Mehdi Elkourchi, Zakaria Chahbi, Said Adnor, Soukaina Wakrim

**Affiliations:** 1 Service de Radiologie, Centre Hospitalier Universitaire Agadir, Faculté de Médecine et de Pharmacie, Université Ibn Zohr Agadir, Agadir, Maroc

**Keywords:** Ischémie mésentérique aiguë, thrombose mésentérico-porte, COVID-19, tomodensitométrie, rapport de cas, Acute mesenteric ischaemia, mesenteric-portal vein thrombosis, Covid 19, computed tomography (CT), case report

## Abstract

L'ischémie mésentérique aiguë (IMA) résulte d'une diminution ou d'une interruption brutale du flux sanguin mésentérique ayant pour conséquence un apport sanguin inadéquat au tractus gastro-intestinal, responsable de lésions ischémiques et inflammatoires évoluant souvent vers une nécrose en l'absence de traitement adapté, l´insuffisance vasculaire peut résulter d'une embolie ou d'une thrombose artérielle ou d'une thrombose veineuse. Nous présentons un cas rare d´ischémie veineuse mésentérique chez un homme de 33 ans dû au coronavirus (COVID-19) chez qui le diagnostic était fait grâce à l´échographie et surtout à la tomodensitométrie (TDM).

## Abstract (en)

Acute mesenteric ischemia (AMI) is due to a sudden decrease or interruption of mesenteric blood flow resulting in inadequate blood supply to the gastrointestinal tract. This causes ischemic and inflammatory lesions often progressing to necrosis in the absence of appropriate treatment. Vascular insufficiency may arise as a result of embolism or arterial thrombosis or venous thrombosis. We here report a rare case of mesenteric venous ischemia caused by coronavirus disease 2019 (COVID-19) in a 33-year-old man in whom diagnosis was based on ultrasound and, in particular, on computed tomography (CT).

## Introduction

L'ischémie mésentérique est une pathologie fréquente, aux étiologies variées et dont les conséquences peuvent aller de la simple altération transitoire de l'activité intestinale à la nécrose transmurale, les thromboses veineuses sont plus rares (5% des cas d´ischémie mésentérique), elles sont parfois peu symptomatiques mais peuvent également être létales, la présentation clinique est non spécifique, d´où le rôle de l´imagerie multimodale qui permet de confirmer le diagnostic. Nous rapportons un cas de thrombose mésentérico-porte et splénique responsable d´une ischémie mésentérique aiguë chez un homme âgé de 33 ans infecté par le coronavirus (COVID-19).

## Patient et observation

**Informations relatives aux patients (présentation du patient)**: nous rapportons le cas d´un homme adulte âgé de 33 ans, admis aux urgences pour douleurs abdominales diffuses d´apparitions brutale et évoluant depuis une semaine. Le patient est tabagique chronique 7 PA, sans antécédents pathologiques particulier, sans notion de prise médicamenteuse ou de plantes médicinales.

**Résultats cliniques**: à l´examen clinique, on retrouve un patient conscient (SG 15/15), normocarde (FC 80 battements/min), normotendu et légèrement tachypnéique (FR 22 Cycles/min), apyrétique (36°). Une défense abdominale diffuse à était retrouvée avec un silence à l´auscultation abdominale sans autres signes pathologiques particuliers.

**Chronologie**: le début de la symptomatologie remonte à une semaine par l´installation de douleurs abdominale brutales au début localisée et rapidement généralisées à tout l´abdomen.

**Figure 1:**
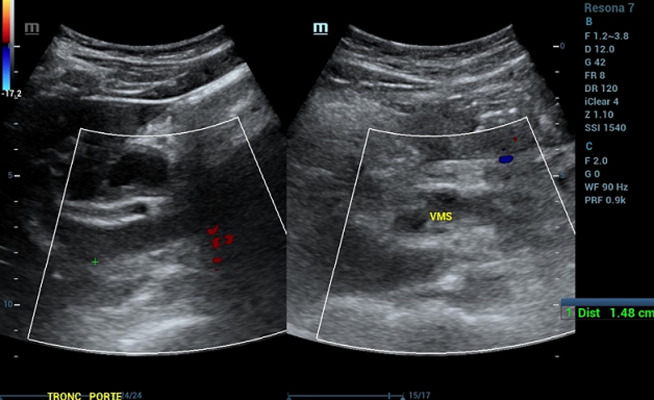
échographie abdomino-pelvienne, tronc porte et VMS imperméables

**Figure 2:**
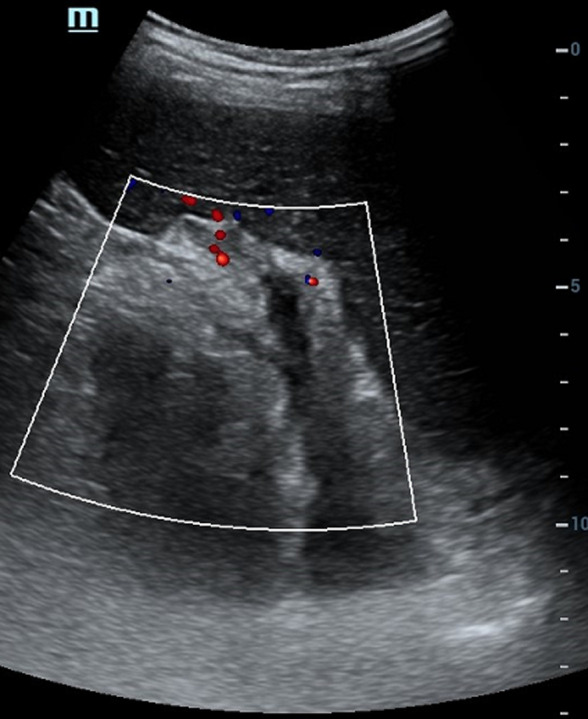
échographie abdominale, veine splénique imperméable

**Figure 3:**
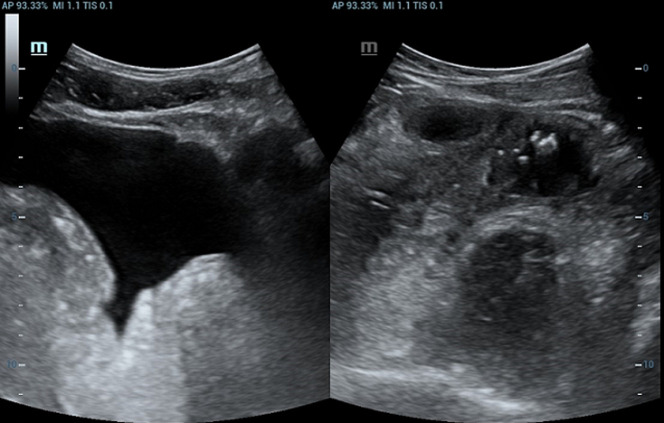
échographie abdominale, épanchement intrapéritonéal

**Figure 4:**
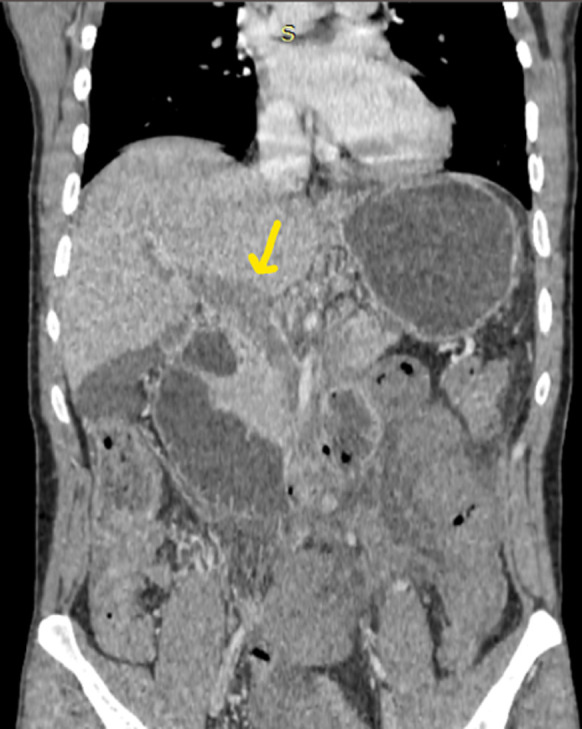
tomodensitométrie (TDM) abdominale en coupe axiale avec reconstruction coronale avec injection de PDC au temps veineux objectivant une thrombose veineuse du tronc porte

**Figure 5:**
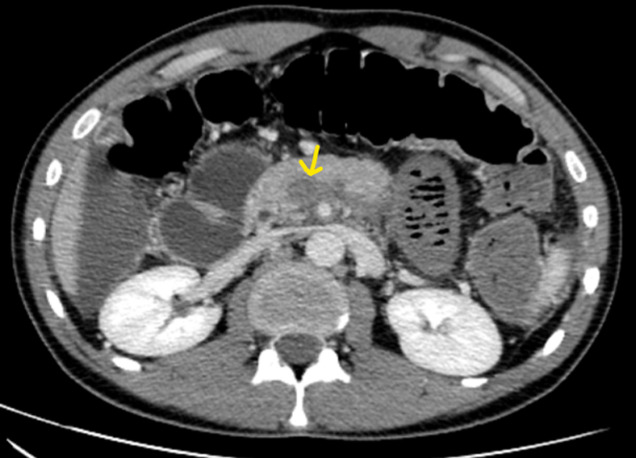
tomodensitométrie (TDM) abdominale en coupe axiale avec injection de PDC au temps veineux, thrombose de la VMS

**Figure 6:**
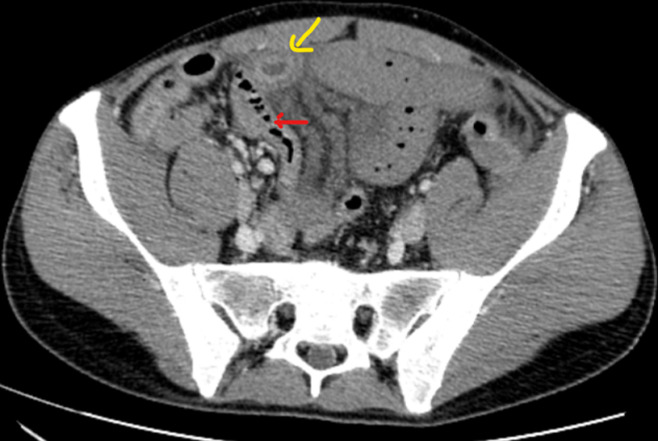
tomodensitométrie (TDM) abdominale en coupe axiale a avec injection de PDC au temps veineux, œdème sous muqueux (flèche jaune) pneumatose pariétale (flèche rouge)

**Démarche diagnostique**: en ce qui concerne le bilan biologique, il a objectivé la présence d´une hyperleucocytose à 37 000 /mm^3^, une protéine C-réactive (CRP) à 345 mg/L et des D-DIMERES à 7667 ng/mL. Le patient a été testé positif au SARS-CoV-2 via la Réaction en chaîne par polymérase (PCR). Le reste du bilan biologique et revenu normal. L´échographie abdominale a mis en évidence la présence d´un épaississement digestif grêlique diffus associé à un épanchement intrapéritonéal de faible abondance, avec une imperméabilité du tronc porte et de la veine splénique n´ayant pas un signal au doppler couleur (Figure 1, Figure 2, Figure 3). Nous avons complété le bilan par un scanner abdomino-pelvien avant et après injection de produit de contraste (PDC) (Figure 4, Figure 5, Figure 6). Il a objectivé la présence d´un: a) aspect spontanément dense du tronc porte, de la veine splénique, des veines mésentériques supérieur et inferieur et de la veine rectale avec défaut de rehaussement de ces dernières après injection du PDC au temps veineux. b) épaississement circonférentiel régulier du jéjunum associé à de l´œdème sous muqueux et pneumatose pariétale avec absence de rehaussement de la paroi après injection du PDC. c) épanchement intra péritonéal de moyenne abondance. Le diagnostic de l´ischémie mésentérique aiguë sur thrombose veineuse a été retenu.

**Intervention thérapeutique, suivi et résultats**: le patient était mis sous traitement anticoagulant et a bénéficié d´une résection chirurgicale de l´iléon avec une surveillance clinique et biologique. L´évolution était marquée par l´installation du syndrome du grêle court secondaire avec aggravation de l´état du patient et décès.

## Discussion

L'ischémie mésentérique aiguë (IMA) est une urgence abdominale mortelle définie par l'association d'une insuffisance vasculaire mésentérique et de lésions ischémiques intestinales. La pierre angulaire du diagnostic est le scanner injecté multiphasique, en plus de permettre un diagnostic correct, il joue un rôle pronostique. En effet, l'IMA est une étape vers la nécrose irréversible, la défaillance d'organe et la mort et doit être identifiée tôt afin d'être prise en charge en conséquence. La défaillance d'organe, l'élévation du taux sérique de lactates et la présence de signes de nécrose en imagerie sont les trois signes principaux qui aident à différencier les formes précoces des formes tardives. Un rehaussement absent ou diminué de la paroi intestinale, une dilatation des anses, une pneumatose pariétale surtout avec aéroportie ou des bulles de gaz extradigestives sont des signes tomodensitométriques majeurs car ils sont associés avec une sévérité croissante à la nécrose pariétale [1].

La thrombose veineuse mésentérique représente environ 5 à 15 % des causes d'IMA [2], le pronostic est mauvais avec un taux de mortalité de 95 % sans traitement, tombant à environ 70 % lorsque le traitement chirurgical est effectué [3]. Elle constitue la troisième localisation thrombotique après les atteintes pulmonaires et des membres, à égalité avec les thromboses cérébrales [4]. L´occlusion d´origine veineuse peut être consécutive à de multiples maladies avec état pro-thrombotique [5]. Dans 10% des cas environ, la thrombose veineuse mésentérique est idiopathique, aucune cause n'étant retrouvée. Le COVID-19 est une nouvelle maladie infectieuse dans la famille des coronavirus, causant le plus souvent des atteintes respiratoires importantes. Les symptômes classiques du virus SARS-CoV-2 comprennent la fièvre, la toux, et la dyspnée. Un nombre croissant d´études ont démontré des manifestations extra-pulmonaires de COVID-19 d´ordre hématologique, cardiovasculaire, rénale, gastro-intestinale et hépatobiliaire, systèmes endocriniens, neurologiques, ophtalmologiques, et dermatologiques [6].

Une méta-analyse récente de Ye *et al*. rapporte que des manifestations gastro-intestinales surviennent chez environ 3 à 40,7% des patients, les symptômes les plus courants étant la diarrhée, l'anorexie, nausées, vomissements, douleurs abdominales, éructations, distension abdominale et gastro-intestinale hémorragie. La diarrhée était le symptôme gastro-intestinal le plus fréquemment signalé, survenant chez 2 à 38,1% des patients infectés. L'anorexie était présente chez 9,3 à 66,7% des patients infectés. Les patients éprouvant des nausées et des vomissements représentaient 1à 19,5%. Des douleurs abdominales ont été signalées chez 1,2 à 19,1% des patients [7].

Selon Goldberg-Stein *et al*. la douleur abdominale était l'indication la plus courante chez les patients qui ont eu un scanner abdomino-pelvien et c'était le symptôme le plus courant associé à des résultats positifs à la TDM. Environ 57% des examens tomodensitométriques abdominopelviens réalisés sur des patients avec COVID-19 ont été pathologiques, et c´était le plus souvent des anomalies du tractus gastro-intestinal (31%). Parmi ces anomalies gastro-intestinales, l´épaississement mural était le plus souvent retrouvé [8]. Les signes radiologiques fréquemment décrits dans le cadre de l'imagerie abdominale chez les patients COVID-positifs avec des symptômes gastro-intestinaux sont les épaississements de la paroi intestinale, la dilatation colique à contenu liquidien, la pneumatose, le pneumopéritoine, l´invagination, la thrombose vasculaire, l´ischémie mésentérique et l´ascite [9]. La fréquence de ces manifestations met en évidence la nécessité de rechercher et d'évaluer les autres sites et localisations du COVID-19 au même ordre que la localisation pulmonaire.

Bien qu'il ne soit pas complètement compris, le mécanisme des manifestations gastro-intestinales est probable multifactorielle et plusieurs mécanismes ont été mis en évidence. Le SARS-CoV-2 pénètre dans les cellules hôtes via la liaison du récepteur de l'enzyme de conversion de l'angiotensine 2 (ACE2) et de la sérine protéase TMPRSS2, qui s'expriment principalement dans le système respiratoire, mais aussi dans l'intestin grêle, l'œsophage, et le côlon [7]. Il y a eu identification d´une protéine de la nucléocapside virale dans les cellules épithéliales gastriques, duodénales, les cellules épithéliales rectales et les cellules glandulaires entérocytes ainsi que l'ARN viral dans les selles. L'examen histopathologique a permis de visualiser l´inflammation endothéliale des vaisseaux sous-muqueux intestinaux avec ischémie mésentérique, évoquant une des lésions vasculaires. Des lésions tissulaires inflammatoires ont également été évoquées en raison de l´identification des plasmocytes, des lymphocytes et de l'œdème interstitiel dans l'estomac, le duodénum et le rectum. On pense que l´inflammation sévère joue un rôle dans l'hypercoagulation: condition observée au cours de la maladie à coronavirus [10].

Les manifestations intestinales du COVID-19 comprennent l´inflammation intestinale, l´occlusion intestinale, l´iléus, l´ischémie intestinale et l´invagination. Khader *et al*. ont présenté un cas de colite du caecum et du côlon droit chez une femme en bonne santé infectée par le SARS-CoV-2 [11]. De même, Sattar *et al*. ont présentés deux cas de colite, le premier patient avait une atteinte de tout le cadre colique ainsi que le sigmoïde, et l'autre patient avait une atteinte de la partie proximale du côlon transverse. Ils présentent également un cas unique d'iléus colique avec des bulles d´air dans la paroi intestinale [12]. L´inflammation intestinale est une constatation courante chez les patients infectés par le SARS-CoV-2 qui présentent une douleur abdominale non spécifique. Nous présentons un patient atteint d'ischémie veineuse mésentérique et d'infarctus dû au SARS-CoV-2, une manifestation gastro-intestinale mal décrite dans la littérature. O´Shea *et al*. évalué les aspects radiologiques de la coagulopathie associée au COVID-19. Leur étude a révélé que 26% des patients atteints de COVID-19 qui ont eu une imagerie présentaient des signes radiologiques en faveur de cette coagulopathie induite par le coronavirus. Les résultats les plus courants étaient l'embolie pulmonaire (EP) et les thromboses veineuses; cependant, l´ischémie intestinale et l´infarctus étaient présents chez 11% des patients souffrants d´une coagulopathie associée à une infection au coronavirus. Trois de ces patients avaient de multiples localisations associées à l´EP, l´infarctus hépatique ou à l´infarctus du parenchyme cérébelleux. Des thromboses Gastro-intestinal impliquant le système vasculaire mésentérique étaient présentes dans ces cas [13]. Revzin *et al*. présentent également un cas d'ischémie intestinale due à un gros thrombus dans l'artère mésentérique supérieure [14]. Les anomalies de la coagulation dues au SRAS-CoV-2 sont de mauvais pronostic [15]. Alors que la coagulopathie est une constatation courante chez les patients atteints de maladie à coronavirus, l'ischémie mésentérique veineuse reste une étiologie rare des douleurs abdominales comparées aux autres étiologies.

## Conclusion

La thrombose veineuse mésentérique est une étiologie rare de l'ischémie intestinale, et notre patient avait un rare cas d'ischémie veineuse mésentérique et d'infarctus dû à une infection par le SARS-CoV-2. Les manifestations cliniques ne sont pas spécifiques à cette pathologie d´où l´intérêt capital de l´imagerie médicale en matière de diagnostic et thérapeutique. L´atteinte systémique est fréquente et est dû à la fois à l'inflammation gastro-intestinale et à la coagulopathie sous-jacente ce qui impose des investigations approfondies dans la plupart des situations. La prise en charge est chirurgicale dans la plupart des cas. Le pronostic, dépend de l´origine de cette ischémie, de son étendu et de la précocité de prise en charge d´où la nécessité d´un diagnostic précoce.
